# Feasibility of Augmented Reality for Pediatric Giant Supratentorial Tumors: A Report of Three Cases

**DOI:** 10.7759/cureus.56750

**Published:** 2024-03-22

**Authors:** Yilong Wu, Jonis M Esguerra, Sai Liang, Sharon YY Low

**Affiliations:** 1 Neurosurgical Service, KK Women's and Children's Hospital, Singapore, SGP; 2 Neurological Surgery, Vicente Sotto Memorial Medical Center, Cebu, PHL; 3 Neurosurgery, National Neuroscience Institute, Singapore, SGP

**Keywords:** neuro-oncology surgery, 3d model in neurosurgery, neurosurgery, brain tumors cns tumors, healthcare tech contest

## Abstract

Giant supratentorial brain tumors (GSBTs) in children are uncommon and extremely challenging entities unique to pediatric neurosurgery. Factors such as young patient age, need for urgent intervention, intraoperative blood loss, and ongoing raised intracranial pressure symptoms are examples of difficulties faced. Recently, there has been a growing body of literature on augmented reality (AR) in adult neurosurgery. In contrast, the use of AR in pediatric neurosurgery is comparatively less. Nonetheless, we postulate that AR systems will be helpful for understanding spatial relationships of complex GSBT anatomy for preoperative planning in a timely fashion. This study describes our experience in trialing AR as a potential tool for three cases of pediatric GSBTs. Overall, the AR platform offers our neurosurgical team excellent visuospatial insights for preoperative decision-making. However, we observe that substantial time is required to set up the AR system prior to each clinical case discussion by the neurosurgical team. In congruency with existing literature, our preliminary results report that there are still obstacles that need to be addressed before the technology can be seamlessly implemented into the clinical workflow for these time-sensitive childhood brain tumors. To our knowledge, this is the first study to report the potential use of AR for complex pediatric GSBT cases.

## Introduction

Brain tumors are the most common solid tumors in children, comprising 25% of all childhood cancers [1,2]. Conversely, giant supratentorial brain tumors (GSBTs) referring to tumors larger than 5 cm that arise in the supratentorial compartment make up only a small proportion of this group [3-5]. Cumulative studies have highlighted the unique challenges of managing these patients due to their large sizes, heterogeneous histology, and age-related physiology [5-7]. For GSBT surgery, it is well-established that young children are at high risk of operative complications such as profuse bleeding, in addition to other significant postoperative morbidities [3]. Additional factors associated with high morbidity and mortality include delayed diagnosis along with surgical and anesthesia drawbacks in this setting [8]. Historically, GSBTs are believed to be inoperable and the literature on the topic is limited [6]. Separately, modern technology has paved the way for improved operative adjuncts in the neurosurgeon’s armamentarium. In particular, the use of augmented reality (AR) in neurosurgery has been steadily growing [9,10]. This innovative, computer-based platform allows users to overlay visual information in their field of view by combining real and virtual objects in the real environment, running in real-time, and connecting real and virtual objects [11]. Established applications include pre-operative planning of surgical procedures, neuroanatomy education, and so forth [10,12,13]. Specific to GSBTs in young children, we postulate that AR systems will be helpful for understanding spatial relationships of complex brain tumor anatomy in pediatric craniums in a short time. This is important because GSBT surgeries are often time-sensitive and require careful operative approaches to avoid catastrophic neurological injury. This proof-of-concept study is undertaken to investigate the feasibility of using AR as a potential tool for surgical planning for children diagnosed with GSBTs, in corroboration with published literature.

## Case presentation

Overview of study workflow

**Figure 1:**
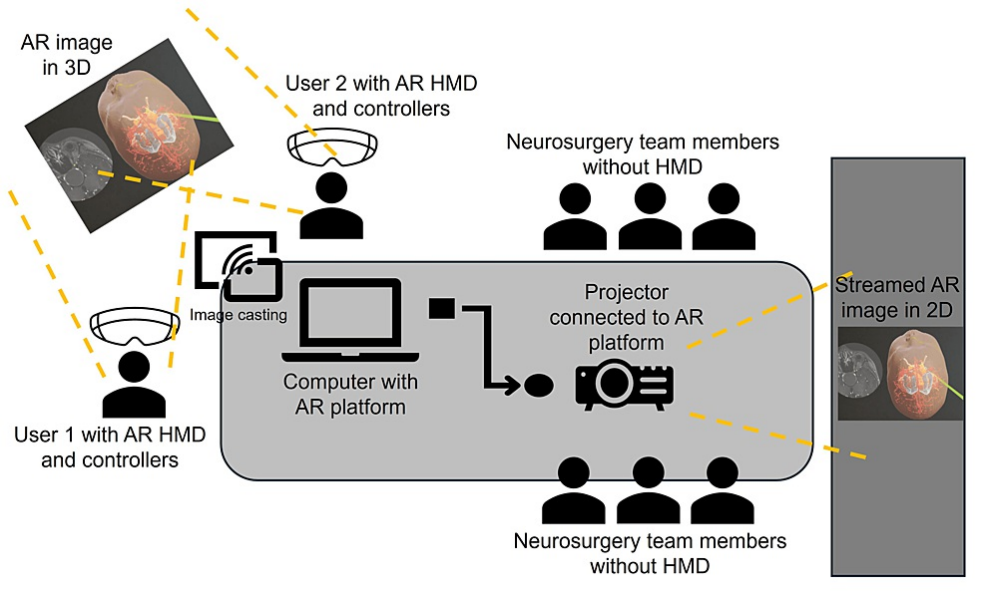
Schematic illustration of the room layout during each case discussion. Two designated users of the augmented reality (AR) system are positioned at one end as physical space is required for them to view and manipulate the three-dimensional (3D) models via their head-mounted devices (HMDs). These models are streamed via the AR platform as two-dimensional (2D) images and projected on a separate, large screen for the remaining members of the clinical team to view and participate in the discussion [Note: some aspects of this figure were created with the help of stock images from Microsoft® Powerpoint® for Microsoft 365 (Microsoft Corporation, Redmond, WA, USA)].

This is an ethics-approved, retrospective study for pediatric brain tumor patients managed by the Neurosurgical Service, KK Women’s and Children’s Hospital (SingHealth CIRB Ref: 2014/2079). The primary aims of this study include firstly, investigating the visuospatial quality of AR systems for preoperative planning of GBSTs and next, assessing its feasibility to be integrated into our current workflow. For this study, previously operated clinical cases of GBSTs are chosen based on their close proximity to critical neurovascular structures and eloquent locations. All selected cases have undergone either gross or near-total resection uneventfully. Briefly, magnetic resonance imaging (MRI) sequences relevant to each patient are uploaded into the Elements Anatomical Mapping (Release 1.0) as part of the BrainLab Elements software (BrainLab AG, Munich, Germany) for auto segmentation, as previously described by others in the literature [14-16]. Tumor boundaries are segmented using the computer-assisted SmartBrush to create a three-dimensional (3D) tumor volume. For specific cases, subcortical white matter tracts are reconstructed from diffusion tensor imaging (DTI) sequences with the Fibertracking function. For the AR aspect of this study, the Magic Leap Mixed Reality Viewer (https://www.magicleap.io/brainlab-mixed-reality-viewer) is used. This is an AR head-mounted device (HMD) that integrates with the BrainLab Elements software to display image reconstructions within a virtual viewing space. Two HMDs are allowed to register to the same virtual environment to enable a shared viewing space. Those in the neurosurgery team without headsets can view the same image reconstruction in two-dimension (2D) streamed on a separate computer screen (Figure 1).

Illustrative case example 1: two-year-old with supratentorial ependymoma

**Figure 2:**
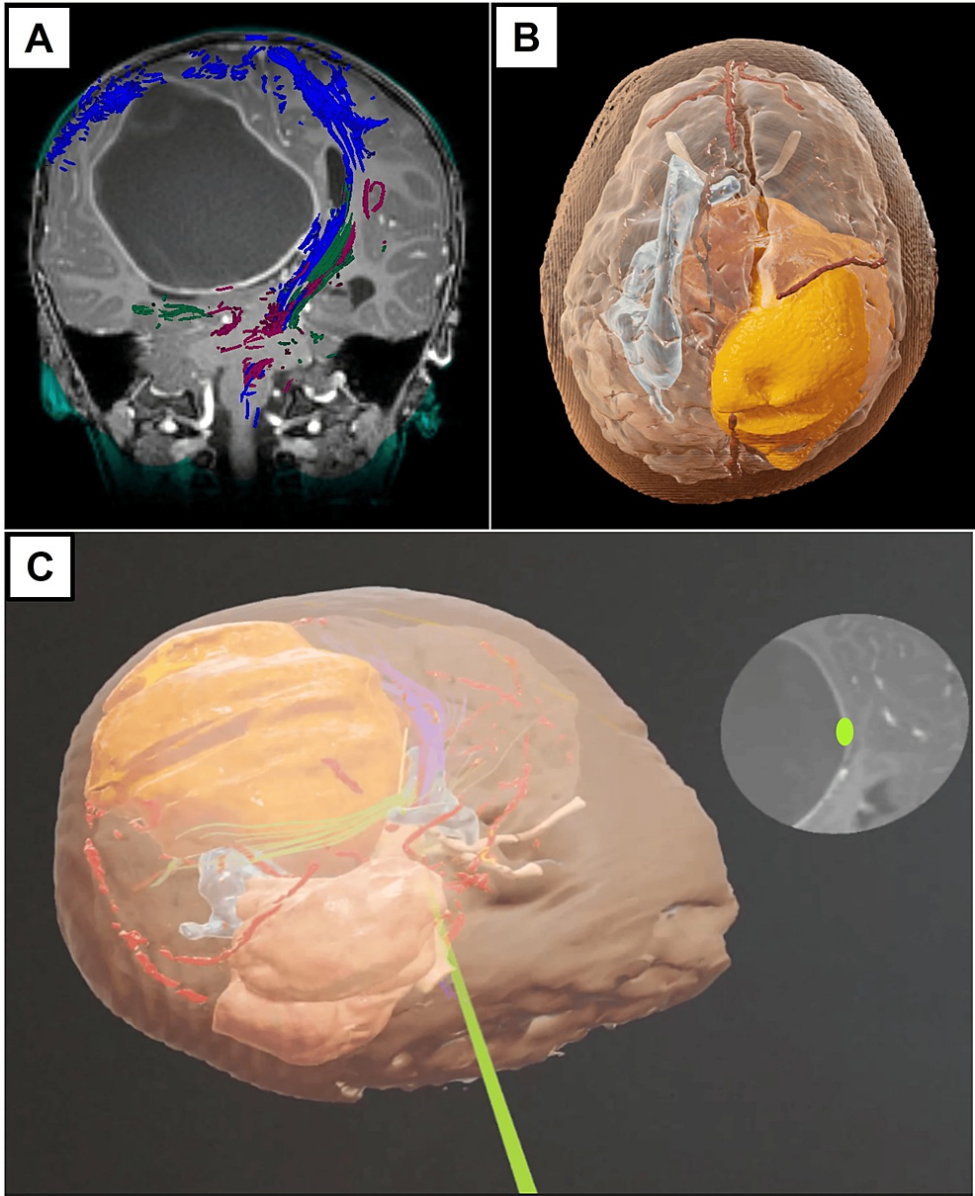
Summary of images used for Case 1. (A) Representative preoperative magnetic resonance imaging (MRI) image in the post-contrast T1-weighted sequence in coronal direction. Here, the right giant supratentorial brain tumor (GSBT) is pushing the adjacent corticospinal (blue), corona radiata (purple), and optic radiation (green) white matter tracts to the left. Of note, tractography is generated via the Modus Plan™ (Synaptive Medical, Toronto, Canada). (B) Representative image of the three-dimensional (3D) model after segmentation of the tumor (yellow-gold) and surrounding normal structures. Here, the tractography is omitted for the purposes of displaying the model’s anatomical details. (C) Representative streamed augmented reality (AR) images placed next to the corresponding MRI brain slide to allow the neurosurgical team to know the location of the brain-tumor interface posterior to the adjacent corticospinal tract (light green).

This is a previously well-toned toddler who presented with motor regression. MRI brain scans reported a 9.7 x 8.1 x 7.1 cm supratentorial solid-cystic intrinsic tumor in the right high parietal lobe. DTI sequences showed that the left corticospinal tract was anterior to the tumor. In this case, the aim was to plan a surgical trajectory to avoid injury to the motor cortex next to the tumor (Figure 2). 

Illustrative case example 2: three-year-old with choroid plexus carcinoma

**Figure 3:**
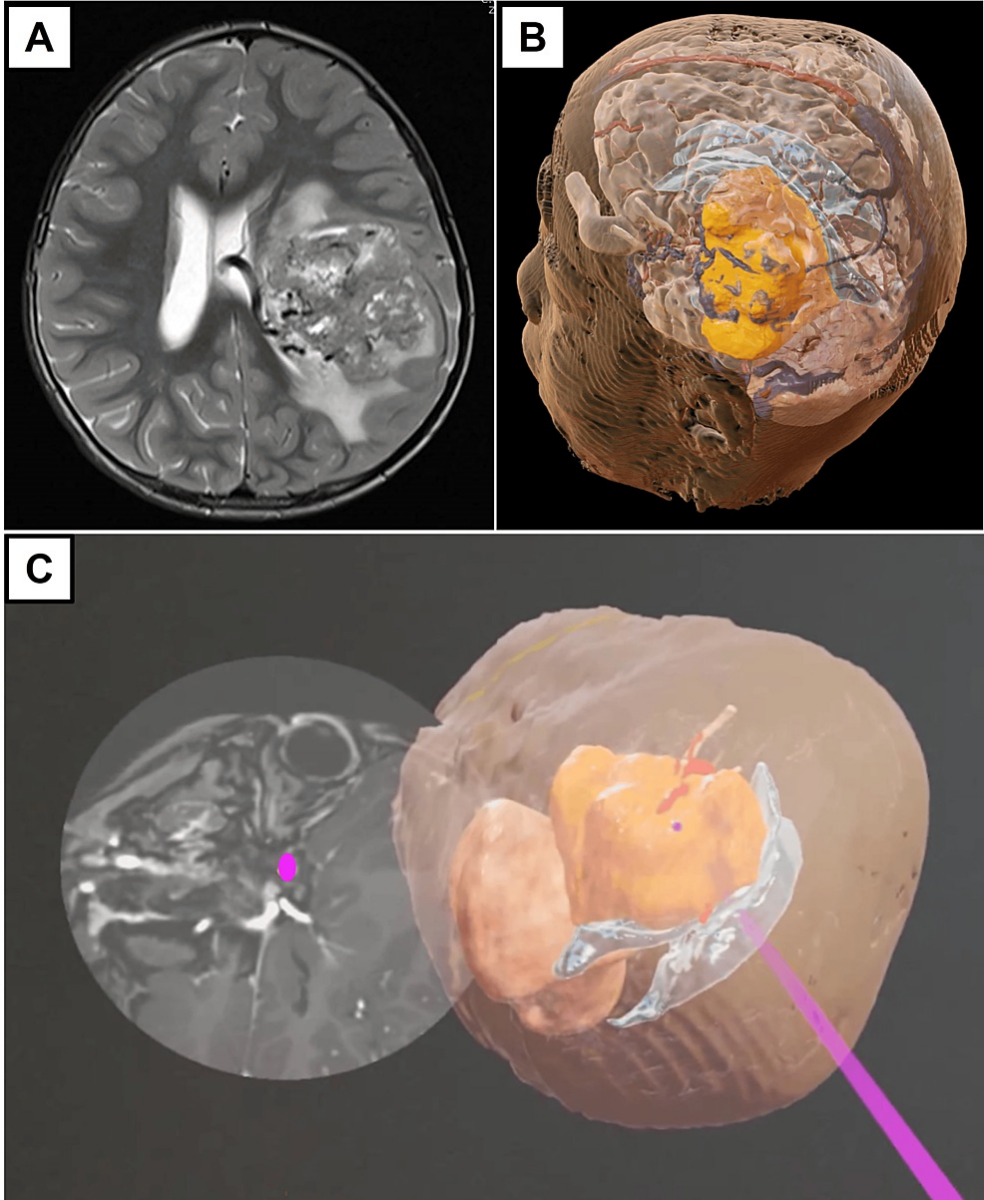
Summary of images used for Case 2. (A) Representative preoperative magnetic resonance imaging (MRI) image in the T2-weighted sequence depicting a highly vascular giant supratentorial brain tumor (GSBT). (B) Representative image of the three-dimensional (3D) model after segmentation of the tumor (yellow-gold) and adjacent ventricle and parenchyma. (C) Representative streamed augmented reality (AR) images placed next to the corresponding MRI brain slide to allow the neurosurgical team to know the location of the feeding vessels (red) in relation to the tumor (magenta).

This patient presented with a history of new-onset right eye squint associated with vomiting. Clinical examination demonstrated anisocoria of the right eye and bilateral papilledema. Follow-up MRI brain reported a 5.6 x 6 x 5.8 cm heterogeneously enhancing mass spanning the left frontal, temporal, and parietal lobes with perilesional edema and midline shift. Angiographic sequences showed feeding arteries from the left middle cerebral artery and an enlarged draining vein on the superomedial aspect of the mass draining into the left internal cerebral vein. On the arterial phase on dynamic imaging, there was a visualization of the same draining vein and perfusion in the mass, indicating the presence of intratumoral shunts. In this case, the main surgical concern was the high vascularity of the tumor in the context of a young child (Figure 3). 

Illustrative case example 3: 11-year-old with craniopharyngioma

**Figure 4:**
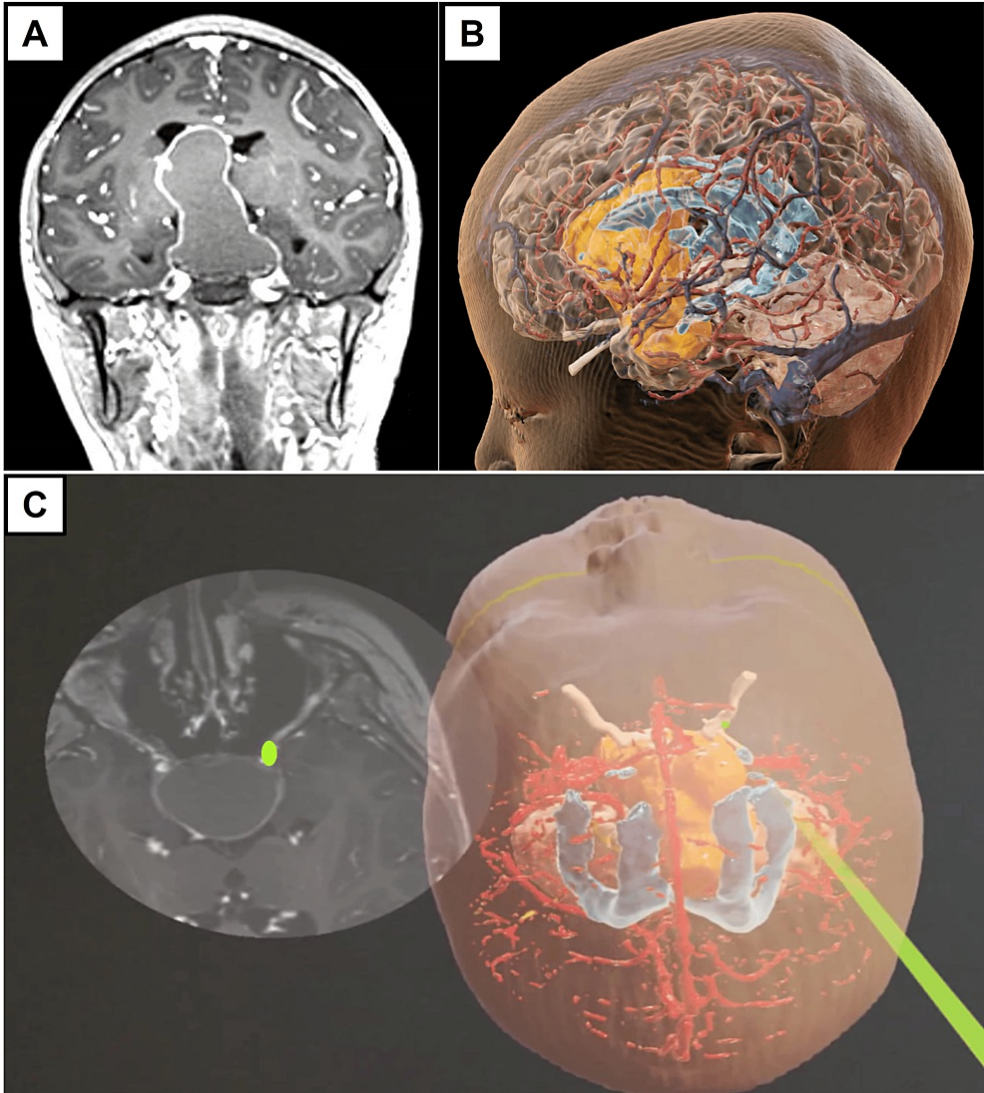
Summary of images used for Case 3. (A) Representative preoperative magnetic resonance imaging (MRI) image of the sellar-suprasellar giant supratentorial brain tumor (GSBT) in the post-contrast T1-weighted sequence in the coronal direction. (B) Representative image of the three-dimensional (3D) model after segmentation of the tumor (yellow-gold) and adjacent structures. For this case, the anterior optic apparatus, bilateral internal cerebral arteries, and their branches were of particular interest. (C) Representative streamed augmented reality (AR) images placed next to the corresponding MRI brain slide. Here, the 3D model is placed in the simulated supine position to allow the neurosurgical team to know the location of the right optic nerve (light green) in relation to the tumor.

This patient presented with delayed puberty, worsening visual acuity, and bilateral temporal hemianopia. Urgent MRI brain showed a 6.4 x 4.0 x 2.8 cm sellar-suprasellar mass exerting mass effect on the corpus callosum and pericallosal cistern. Of concern, the optic chiasm, bilateral optic nerves, and optic tracts were compressed and not well seen. In this case, the aim was to decompress the anterior visual apparatus emergently while preserving the adjacent internal cerebral vessels and their branches (Figure 4). 

Quantitative time required for inclusion of the AR infrastructure setup

**Figure 5:**
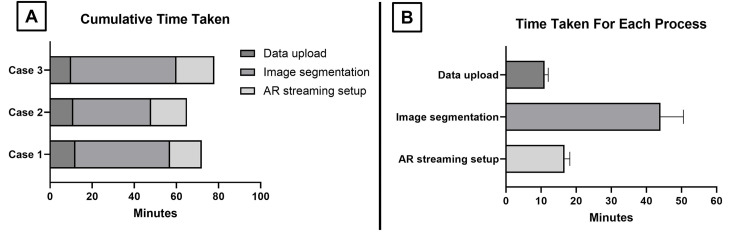
Summary of quantitative time required for the setup of the AR system. (A) Graph depicting cumulative time with breakdown of individual processes for each clinical case. (B) Graph showing mean with standard deviation (SD) for the time periods required for each part of the AR setup. AR: Augmented reality

The time required for setup and subsequent use of the AR system was documented in three parts: data upload, image segmentation, and AR streaming setup. As this aspect of the study aim was focused on quantifying how much additional time would be needed to integrate the AR setup into our usual workflow, time spent on the actual clinical case discussion was excluded. To avoid the bias of a learning curve, two designated members of the neurosurgical team were trained to use the equipment, software, and platform to offset potential delays. We observed the following: data upload, mean = 11 (± 1.0) minutes; image segmentation, mean = 44 (±6.6) minutes; AR streaming setup, mean = 16.7 (±1.5) minutes; and cumulative time for AR set up per case, mean = 71.7 minutes. Graphs and descriptive statistics are generated using GraphPad Prism Version 10.2 (GraphPad Software, La Jolla, CA, USA) (Figure 5). 

## Discussion

Neurosurgery plays an important role in the treatment of pediatric GSBT despite its immense challenges [6]. Factors such as young patient age, life-threatening symptoms of raised intracranial pressure, heterogeneity of tumor types, intraoperative blood loss, and the risk of poor long-term outcomes are considerable difficulties faced by clinicians. Furthermore, these tumors are often overlooked in the early phases due to non-specific or even absence of signs and symptoms in the very young, resulting in delayed presentation [17]. At the time of diagnosis, surgery is time-sensitive owing to high risks of neurological deterioration. Most published literature is limited to small retrospective case series [5,6,18,19]. To date, there are no established guidelines with regard to the optimal management of GSBT [20]. Nonetheless, the core principles of maximal safe resection on the premise of alleviating raised intracranial pressure, protecting the structure of surrounding normal brain parenchyma, and preserving neurological function still apply. Under such circumstances, a clear understanding of the anatomical relationship between pathology and critical intracranial structures is expected of the neurosurgeon [21]. This is especially relevant in GSBTs, whereby visualization of tumor interfaces, eloquent brain regions, and neurovasculature, are often obstacles to planning safe and efficacious approaches in pediatric patients.

Presently, there is extensive literature on mixed reality (MR) technology in the adult neurosurgery setting [16,22-24]. In contrast, similar applications for children are comparatively less [25]. Briefly, a mixed reality system encompasses two similar yet distinct systems: virtual reality (VR); and augmented reality (AR) [5]. The former (i.e., VR) immerses users in an artificial digital environment, while the latter (i.e., AR) has the capability to overlay virtual objects in the real-world environment [25]. To be clear, AR systems have been in existence since the 1960s. Early applications for this technology include military and pilot training [26]. In recent years, advancements in both hardware and 3D software have helped to demonstrate the potential of AR for surgical use. Most significantly, AR has the capability to merge virtual intra-operative content with elements of the actual operating theatre [27]. Furthermore, AR devices allow neurosurgeons to use individual HMDs to build 3D virtual models to select the surgical approach, design the incision, anticipate the possible intraoperative conditions, measure the tumors volumetrically, and examine surrounding structures; hence, paving the way for direct and clear guidance for the operation plan [28]. To date, findings from various systemic reviews on AR’s role in neurosurgery training have been largely positive; therefore, reaffirming its use in neurosurgery education [27,29,30]. Nonetheless, the next step forward to implement AR as a routine clinical application is still in the making. In this study, we explore the use of AR’s purported excellent visuospatial details of selected intracranial structures of interest in our cohort of GSBT patients and assess the potential of its applicability in surgical planning for these complex cases. At this point, we reiterate that the limitations of other existing neurosurgical adjuncts have been previously discussed and beyond the context of this paper [25].

For the most part, our results suggest that the use of AR for GSBT provides the operating neurosurgical team with increased 3D spatial awareness of specific intracranial structures that we endeavor to preserve during the course of surgery. Following that, the platform’s visual benefits facilitate in-depth discussions amongst the team with regard to neuroanatomical understanding and perioperative planning of the surgery. However, the extra time required for the image segmentation, equipment calibration, streaming of images and individual user learning experience delays our clinical workflow. In particular, considerable time had to be spent on manually segmenting anatomical structures of interest, especially those that were compressed, distorted, or shifted by the GSBTs. Furthermore, efforts to address device adjustment issues during real-time interaction between virtual and reality by the users led to periods of delay during our case discussions. We note that similar concerns have been previously highlighted by other studies focused on AR applications for neurosurgery [28,31,32]. Specific to GSBT cases, the additional time required for setup does not make it feasible to be used for emergency cases at this stage. Nevertheless, efforts are ongoing to explore the use of the same AR platform for less urgent neurosurgical cases.

Overall, the current evidence indicates existing pitfalls need to be resolved before the actual implementation of AR into routine clinical use [9,25,32,33]. For instance, integrating new medical technology into existing healthcare systems is a significant undertaking. High expenses incurred from the purchase and maintenance of the technology in question are likely to contribute to rising healthcare costs [34]. Moreover, the cost-effectiveness of AR systems in the clinical setting remains untested at this juncture [35]. To offset costs incurred by new infrastructure, the ideal situation will be for the chosen technology to have other uses within the hospital setting by other healthcare professionals. Some studies have explored the use of MR modules for pre-operative counseling to improve patients’ understanding and reduce their anxiety about their pending inpatient experience [36]. Concurrently, the same technology has been shown to improve patients’ understanding of their own disease and hence, empower them to adhere to treatment [37]. In addition, the use of MR platforms in neurosurgical education is already well-established [26,27,38]. For the trainees, simulation emulates a real-life scenario that allows them to practice procedural skills in a safe environment [39,40]. Others have taken a step further by implementing the use of AR in the operating theatre (OT) for real-life neurosurgery cases [12,16,41]. However, many of them report ongoing difficulties faced such as intra-operative brain tissue movement in real-time affecting planning accuracy [42,43]. Under such circumstances, the static nature of virtual holograms based on preoperative neuroimaging and disturbances in-depth perception by the users are technical hurdles for accurate intraoperative guidance [44]. In the meantime, we concur with previous studies that the implementation of AR systems into clinical workflows is still presently a work in progress.

## Conclusions

We describe our preliminary experience in trialing AR as a potential tool for pediatric GBSTs. Despite the drawbacks that are congruent with published literature, the AR platform offers our neurosurgical team excellent visuospatial insights for preoperative decision-making. Nonetheless, only a large-scale, prospective cohort study will be able to determine the true feasibility of AR technology for these challenging and highly complex tumors in children.
